# Tranexamic acid: single topical application for femoral neck fractures treated with arthroplasty results in lowest blood loss

**DOI:** 10.1007/s00068-024-02675-9

**Published:** 2025-01-21

**Authors:** Kurnoth Anna, Timon Röttinger, Leonhard Lisitano, Nora Koenemann, Stefan Förch, Edgar Mayr, Annabel Fenwick

**Affiliations:** Department of Trauma, Orthopedic, Plastic and Hand Surgery, University Hospital of Augsburg, Stenglinstrasse 2, 86156 Augsburg, Germany

**Keywords:** Blood loss, Proximal femur fracture, Transfusion rate, Tranexamic acid

## Abstract

**Purpose:**

Tranexamic acid is widely accepted for hip fractures but there is no agreement about dose or application method and the use is still off label for hip fractures. The aim of our study was to find the best application method of tranexamic acid in patients with femoral neck fractures comparing total blood loss, hemoglobin and transfusion rate.

**Methods:**

A retrospective single centre cohort study (level I trauma centre) with 2008 patients treated operatively for a proximal femur fracture between January 2016 and January 2022 was performed. 1 g of tranexamic acid was applied in 314 cases (systemic, topic or combined application) if patients consented. Patient data, surgical procedure, complications, and mortality were assessed. Haemoglobin levels, blood loss and transfusion rates were compared amongst application methods.

**Results:**

For 884 femoral neck fractures treated with arthroplasty blood loss was significantly reduced by tranexamic acid which 314 had received in total (1151.0 ml vs 738.28 ml; p < 0.001). 151 patients received 1 g of tranexamic acid systemically which reduced blood loss from 1151 to 943.25 ml. Combined application of 1 g i.v. and 1 g topically reduced blood loss even further to 869.79 ml and topical application achieved the lowest total blood loss at 391.59 ml (average reduction of 759.41 ml compared to without tranexamic acid), p < 0.001. Transfusion rate and amount of RBC units transfused were the lowest for topical use and showed the highest hemoglobin levels postoperatively. Complication rates did not differ for adverse vascular events.

**Conclusion:**

Tranexamic acid effectively reduces blood loss and transfusion rates and shows higher hemoglobin levels postoperatively, without increasing the risk of thromboembolic events after proximal femoral fractures. Single topic application of 1 g for arthroplasty treatment of femoral neck fractures has better results for blood loss reduction than single i.v. or combined application.

## Introduction

Despite improvement in treatment of proximal femur fractures including early surgery and mobilization as well as ortho-geriatric co-management, mortality rates are still up to 30% during the first postoperative year [1, 2]. Prevention methods such as fracture liaison service and osteoporosis treatment are becoming more important, but the incidence of fragility fractures of the hip is still continuously rising [3, 4].

Blood loss is a fundamental perioperative problem in hip fracture, and it has been established that hidden blood loss is much higher than estimated [5, 6] dependent on fracture morphology and medication intake [7, 8]. Major blood loss already occurs at the time of trauma and can cause low hemoglobin at the time of admission [9]. Not only does the resulting postoperative anemia prolong rehabilitation but it often worsens cardiac or renal conditions [10, 11]. Studies have shown postoperative transfusion rates reach 44% [12]. These are linked to high economic costs, longer hospital stays and a significant risk for general or postoperative wound infections [13, 14]. For knee and hip arthroplasty, the infection risk has been proven to increase from 1.74 to 2.88% [15].

Tranexamic acid is an antifibrinolytic drug and inhibits transformation of plasminogen into plasmin and therefore reduces fibrinolysis and stabilizes existing blood clots [16]. The use started for hemorrhage conditions [17] and is expanded to elective arthroplasty. By now numerous studies have proven the efficiency of tranexamic acid for multiple conditions including proximal femur fractures leading to reduced blood loss and decrease of transfusion rate and units [18–20]. Most studies were able to show that the risk for vascular adverse events did not increase [21, 22]. Tranexamic acid now belongs to the world’s most essential medication according to WHO [23].

We performed a first study and were able to show promising results i.e. the reduction of blood loss and transfusion rates after application of tranexamic acid [24]. Many application methods have been described such as local application, single and repeated iv doses or, as recently reported application in A&E directly after admission [25–27]. There is no consensus about the best possible application method. Primarily focusing on the benefit of tranexamic acid itself we now moved on to concentrate on the application method aiming to establish the best application method for tranexamic acid in patients with femoral neck fractures without increasing complication rates and hypothesized the combination of 1 g i.v. and 1 g locally injected tranexamic acid to be the most effective application method.

## Methods

### Data acquisition

We performed a retrospective cohort single centre study (level I trauma centre), level III evidence, coherent with the STROBE statement, extending our previous research cohort to all patients treated operatively for a proximal femoral fracture between January 2016 and January 2022 (former 2020) [24]. All femoral neck fractures as well as per- and subtrochanteric fractures were included. We continued to exclude periprosthetic fractures as well as referrals for revision surgery and polytraumatised patients to avoid bias for other blood loss reasons. Patients without pre- or postoperative labs, with concomitant fractures and patients undergoing further surgical procedures during the first six days after admission for proximal femoral fracture were excluded to avoid false conclusions.

The study conducted was approved by the local Ethics Committee of the University of Regensburg and fulfils the standards of the declaration of Helsinki.

The charts were reviewed for demographic data: age, gender, body mass index BMI, Charlson Comorbidity Index CCI [28] and ASA classification [29], fracture morphology, medication, complications especially thromboembolic events, revisions, labs and blood transfusions. Patients admitted again with a fracture on the contralateral side during the reviewed period were included again as a separate case.

### Therapy

Dependent on pre-operative mobility and comorbidities as well as fracture morphology total or hemi arthroplasty (cemented Müllergeradschaft, Fa Zimmer biomet or uncemented Zweymüllerschaft, Fa Zimmer biomet) was performed for medial femoral neck fractures. Minimal invasive intramedullary nailing PFNa (proximal femur nail antirotation, Fa. Synthes) was performed for pertrochanteric fractures and lateral femoral neck fractures. Subtrochanteric fractures were addressed by open reduction, cerclage and intramedullary nailing in side- positioning [24].

Patients without anticoagulants were treated within 24 h. For patients on direct anticoagulants (DOACs) the last intake was recorded and surgery postponed according to our in-house protocol (renal clearance > 50 ml/min: surgery within 24–48 h; renal clearance < 50 ml/min: surgery 48 h after last intake of DOAC). Postoperatively venous thromboembolism prophylaxis was given from day one with Enoxaparin 40 mg subcutaneously to patients without anticoagulants. Anti-platelet therapy was continued. DOACs and Warfarin were substituted with Tinzaparin-sodium according to patient weight. Warfarin was reversed with Vit K if possible, preoperatively until Quick was > 60%. No prothrombin complex concentrate (PPSB) was given. Neither DOACs nor Warfarin were bridged. Mobilization was initiated from day one after surgery with full weight bearing for all patients.

The blood loss was calculated using the Mercuriali formula [30], based on pre- and postoperative haematocrit and the number of transfused RBCs (Red blood cell) and patients` blood volume calculated by the Nadler formula [31], according to gender and height.

Women: BV (l) = height (m)3_0.3561 + weight (kg)_0.03308 + 0.1833 [26].

Men: BV(l) = height (m)3_03669 + weight (kg)_0.03219 + 0.6041 [26].

Estimated blood loss: BV x (Hct_preop_ − Hct_day 5 postoperative_) + ml of transfused RBC [25].

Haemoglobin levels under 7 g/dl received blood transfusions if consented and between 7 and 8 g/dl transfusions were carried out depending on symptoms and cardiovascular risk factors.

### Tranexamic acid

Tranexamic acid protocols were introduced mid-2018 and all charts were checked for administration of tranexamic acid. 1 g Tranexamic acid was administered in the operating room (OR) if patient was eligible, either systemically or directly into the surgical site at the end of the procedure before closing up or combined locally and intravenously. Since 2018 drains have not been used any more except for revision surgery. If tranexamic acid was applied into the surgical site, no drains were inserted. As tranexamic acid for proximal femur fractures is still off-label use strict exclusion criteria were introduced consisting of the known contraindications despite a possible bias: pulmonary disease including pulmonary hypertension, myocardial infarction, deep vein thrombosis or coagulopathy, stroke or pulmonary embolism in patient history or a high thromboembolic risk [24]. Consent for off label use was taken written.

### Statistical analysis

Statistical analysis was carried out with IBM SPSS Statistics (version 27; IBM Deutschland Ltd., Ehningen, Germany). Normal distribution of all data was verified (Shapiro wilk test). The student’s t-test, chi square, ANOVA variance and binary logistic regression were used to determine differences and influencing factors regarding complications and mortality; 95% confidence intervals and standard deviations were calculated. For data without normal distribution the Wilcoxon Rank Test was used. The significance level was set at 5% (α = 0.05).

## Results

### Demographic data

2008 patients with available labs were included in the study of which 68.5% were female and 31.5% male with an average age of 80.65 years (range 18–103; SD 11.09). 924 femoral neck fractures, 948 pertrochanteric fractures and 136 subtrochanteric fractures with 884 total- or hemiarthroplasty, 949 intramedullary nailing with closed reduction and 132 with open reduction, 38 dynamic hip screws and 5 screw osteosynthesis were included. The mean BMI was 24.36 kg/m^2^ (range 13.5–66.4 kg/m^2^, SD 4.46). The average length of hospital stay LHS was 14.5 days. Surgery was performed 24.87 h after admission (range 0.95–166 h; SD 18.5). The mean CCI was 5.79 points and was slightly higher in the group of patients without tranexamic acid (5.87 points). 1007 patients (50.1%) had anticoagulants upon admission. 54.6% of all patients had no level of care prior to admission and 44.7% were mobile without any kind of aid. 26.5% of the total cohort were able to go home postoperatively and 38.8% were discharged to rehabilitation. There were no statistical differences between the groups.

### Tranexamic acid

**Fig. 1:**
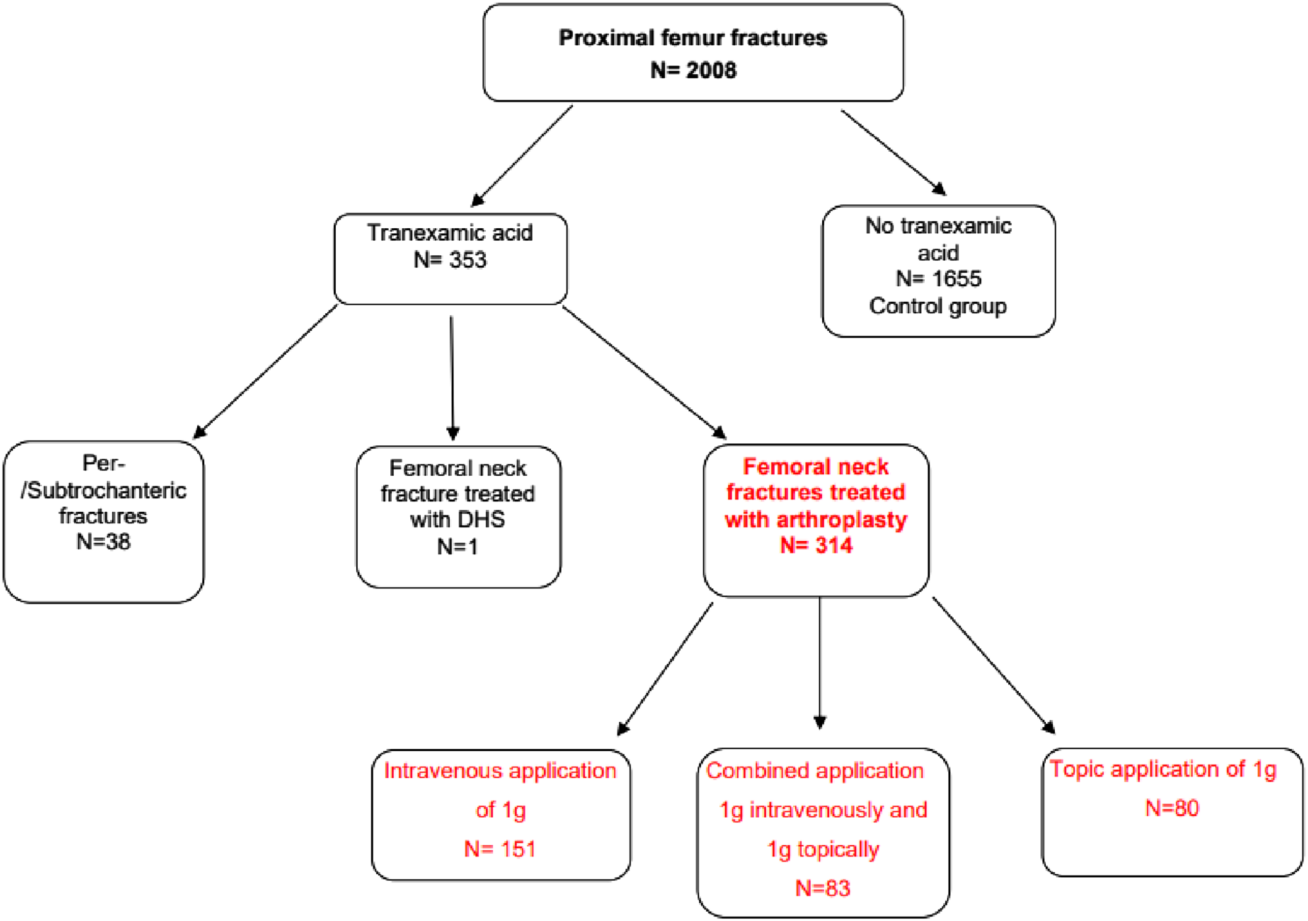
Distribution of patients and application of tranexamic acid as flow chart

353 patients (17.6%) administered tranexamic acid were compared to 1655 patients without tranexamic acid. 190 patients had 1 g intravenous Tx acid, 80 patients received 1 g local application and 83 were given a combination of 1 g intravenously and 1 g locally applied Tx acid. This included 17 pertrochanteric and 21 subtrochanteric fractures, which only received 1 g intravenously. All 38 patients were treated by nail osteosynthesis (19 closed reductions and 19 open reductions + cerclage). 315 femoral neck fractures were administered tranexamic acid (i.v.: n = 152; local: n = 80; combined: n = 83), Fig. 1. 161 patients on anticoagulants were given tranexamic acid.

### Blood loss and Transfusion rates for all proximal femur fractures

**Fig. 2:**
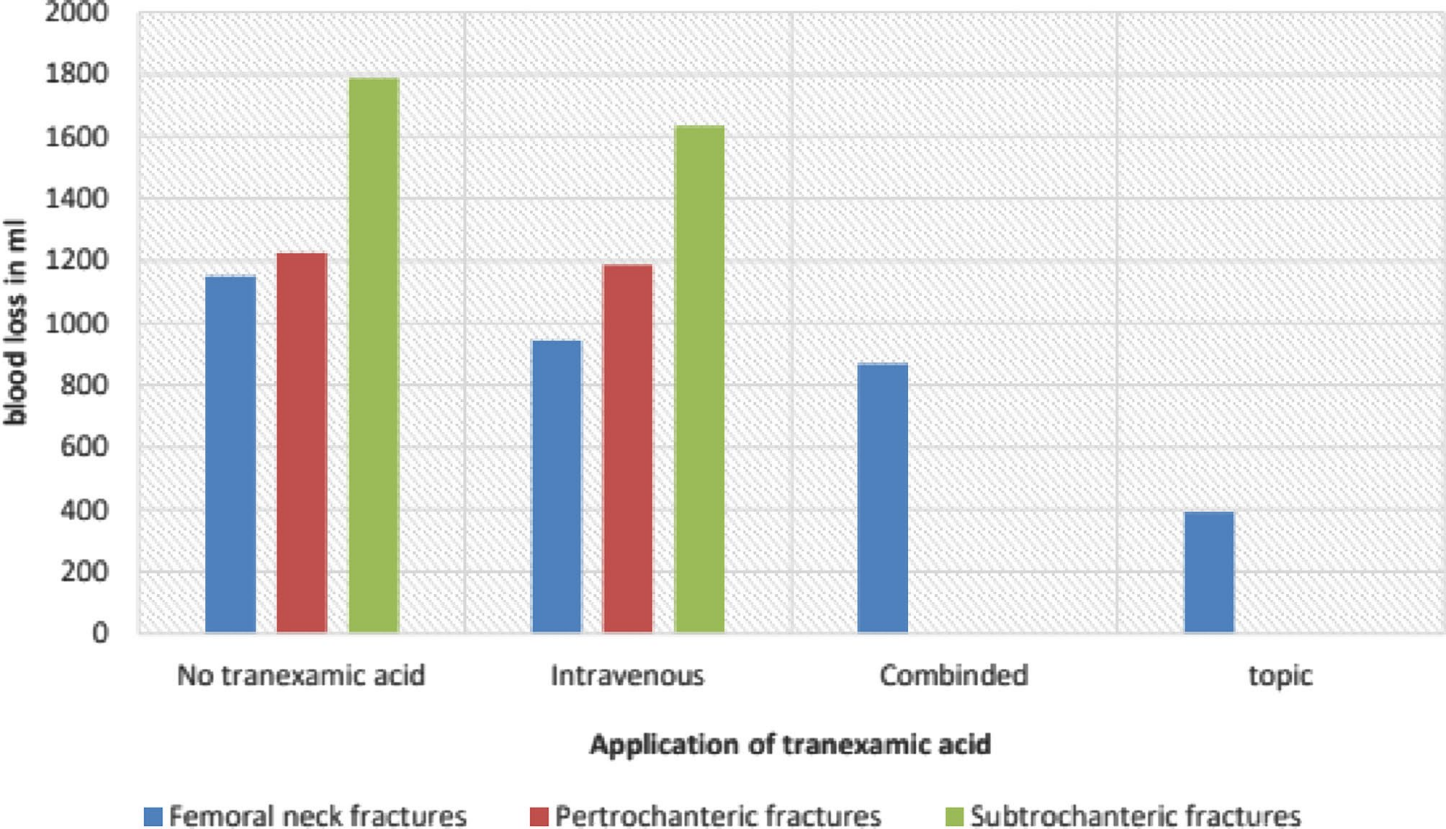
Average blood loss for all hip fractures compared to only femoral neck fractures and application of tranexamic acid

Preoperative hemoglobin did not differ amongst all groups at an average level of 12.31 g/l (range: 4.1–18.8 g/l, SD: 1.8). Hematocrit at admission was 36.38% (SD 5%). Postoperative hemoglobin (day 5) was higher for patients with tranexamic acid (9.77 g/l) than without (9.35 g/l), p < 0.05). The total blood loss of 1211.67 ml (SD 863.6) for all patients without tranexamic acid was significantly higher than after the application of Tx acid. Systemically applied tranexamic acid led to a reduction of blood loss to 1054.85 ml (SD 756.5) but the difference was not significant, p < 0.117. The direct application of 1 g tranexamic acid into the wound led to the largest drop of blood loss to 391.59 ml (SD 314.3), p < 0.00 and the combined application method reduced the total blood loss to 869.79 ml (SD797.2), p < 0.00. The transfusion rate for patients without tranexamic acid was 31.8% (N = 527) and a total of 1055 RBCs were transfused, with one patient requiring 11 RBC units. There was a significant decrease of necessary transfusions after application of tranexamic acid to 18.7% (N = 66) and only 123 RBCs. The maximum RBC units for one patient was 5. Systemic tranexamic acid had a transfusion rate of 23.2% (N = 44), local application led to a transfusion rate of 17.5% (N = 14) and combined use to the lowest rate of 9.6% (N = 8). Subtrochanteric fractures showed the highest total blood loss (1660.36 ml) followed by pertrochanteric fractures with 1228.1 ml and neck fractures with a significantly lower blood loss of 994.82 ml, p < 0.001. In both per- and subtrochanteric fractures there was a reduction of blood loss by 37.61 ml and 153,66 ml. But in both cases the difference after 1 g intravenous Tx acid was not significant whereas femoral neck fractures profited most for tranexamic acid with 367.72 ml less blood loss, p < 0.001, Fig. 2.

### Comparison of application methods—intravenous, topic or combined use for femoral neck fractures

As all 3 application methods were only applied for femoral neck fractures and arthroplasty further analysis into the three methods was carried out with only femoral neck fractures. Within this subgroup of 884 femoral neck fractures treated with arthroplasty blood loss was significantly reduced by tranexamic acid which 314 had received in total (1151.0 ml vs 738.28 ml; p < 0.001). 151 patients had 1 g tranexamic acid systemically which led to a reduction of 207.75 ml to an average of 943.25 ml, p < 0.001. By applying 1 g i.v. and 1 g topically there was a further reduction by 281.21–869.79 ml. The topical application achieved the lowest total blood loss at 391.59 ml (average reduction of 759.41 ml compared to without tranexamic acid), p < 0.001. Whereas preoperative hemoglobin did not differ, hemoglobin after tranexamic acid during arthroplasty was higher on the 5th postoperative day (9.5 g/l vs. 9.8 g/l; p < 0.012). Highest hemoglobin was seen after topical application, but the differences were not significant (i.v: 9.828 g/l; combined: 9.841 g/l; topic: 9.842 g/l; p < 0.094).

121 patients of 570 patients without tranexamic acid needed a transfusion (21.2%). Transfusion rate again was significantly lower after administration of tranexamic acid at an overall of 14.9% (N = 47). Single i.v. use led to a transfusion rate of 16.5% (N = 25). The group of combined application showed a transfusion rate of 17.5% and the lowest rate was achieved by topical application at 9.6% (N = 8), p < 0.064. Tranexamic acid not only led to a reduced transfusion rate but also decreased the total amount of transfused RBC units significantly from 235 RBCs to 88 units (i.v.: 44; combined: 22; topic: 23; p < 0.021).

### Complications

The total complication rate was 24.9% (surgical site infection, urinary tract infection, pneumonia, pulmonary embolism, thrombosis, dislocation, fracture). The complication rate was slightly higher for patients with, in comparison to without tranexamic acid (26.6% vs. 24.6%, p < 0.422). Adverse vascular events including venous thromboembolism, pulmonary embolism, heart attack or stroke were not recorded more often after tranexamic acid. Of all complications that were analyzed the only differences were a higher rate of urinary tract infections for the control group (p < 0.045) and wound infection which occurred more often in the tranexamic group (4.8% vs. 2.5%; p < 0.008). The overall in-house mortality rate was 4.7% (N = 95). The mortality rate amongst the patients without tranexamic acid was 4.9% and with tranexamic acid 3.9% and equally distributed between the application methods.

## Discussion

The perioperative use of tranexamic acid has a growing popularity in acute fracture treatment especially those prone to high perioperative blood loss and dysfunctional outcome due to anemia such as fragility fractures of the hip. The efficiency of tranexamic acid has been well proven in an elective setting for knee and hip arthroplasty for reducing blood loss reliably [25, 27, 32–34]. There is less but still steadily growing evidence for the same effect on reduction of transfusion rates for hip fractures caused by more refrains of administering tranexamic acid to vulnerable most multi-comorbid frail patients at high thromboembolic risk. Zufferey et al. [35] were able to show a decrease in transfusion rates of 30% and some studies have indicated tranexamic acid should be part of a standard protocol for hip fractures. In accordance with Khatib et al. [20] we were also able to show higher hemoglobin levels 5 days postoperatively supporting the positive effect of tranexamic acid for femoral neck fractures treated with arthroplasty.

We previously performed our own retrospective study including all proximal femur fractures treated with arthroplasty and intramedullary nailing (closed and open reduction) and were able to see an overall reduction of total blood loss as well as transfusion rate without an increase of adverse vascular events or other perioperative complications [24]. For femoral neck fractures treated with arthroplasty we applied three methods (1 g intravenously, 1 g topically and the combination of both) and found the greatest reduction of blood loss when simply applying 1 g into the wound during closure, followed by the combination of systemic and local application. The aim of this further analysis including more patients with tranexamic acid was to investigate and compare the three mentioned application methods to find the greatest benefit emphasizing femoral neck fractures.

Multiple application methods and dosage options have been suggested starting from single intravenous or topical use and the combination of both, to sequential administration and dosage according to body weight and/or renal function. None of the methods have proven to be the most effective. Whilst some study showed similar results for single i.v. or intraarticular use others showed promising results for sequential dosing [32]. These were contradicted by a study with repeat doses of tranexamic acid which even led to a higher blood loss and higher drop of hemoglobin [36]. A retrospective study by Wilharm et al. [37] showed no noticeable difference at all between receiving tranexamic acid or not. They put it down to too low dosage in accordance with Wang et al. [27] who concluded that tranexamic acid was efficient starting from 10 mg/kg but even more effective at 15 mg/kg when applied as single dose.

We hypothesized that a combination of systemic and topic application in the fracture situation would be more beneficial than the single i.v. use agreeing with results presented by multiple studies for elective knee- and hip arthroplasty [25, 27, 38]. We were able to reproduce the results showing a further reduction by combined application but even better results for single topical use. Whilst we initially put our good results of single topical application down to a small sample size, we now present equal cohorts for combined and single topical application as well as a twice as large group of single systemic input. Surprisingly, we still show the best results for single topic application into the wound with the lowest blood loss overall. These results are backed up by the highest postoperative hemoglobin and the by far lowest transfusion rates. How can these results be explained and why is topic application better than the combination?

Tranexamic acid has a biological half time of 2–3 h and studies show that 1 g intravenously administered tranexamic acid is eliminated by 95% within the first 72 h and remains unchanged [16, 32]. More detailed analysis shows a bolus of mg 10 mg/kg was eliminated without transformation via the kidney within 24 h (30% after than hour) [39]. Some of the first studies evaluating the potency of tranexamic acid analyzed concentrations in different organs and tissue as well as plasma and were able to see a much higher concentration in tissue material and organs and a significant longer duration time of up to 12 h in tissue than in plasma [40]. Whilst an i.v. bolus of 1 g reaches plasma concentrations of > 10 ml/l for 5–6 h [41], intramuscular administration was absorbed quickly reaching highest plasma levels after half an hour [42]. A study on pigs in which an injection of 30 mg/kg of tranexamic acid was given i.v., measured plasmin activities in various body regions for tranexamic acid activity. Again, it took much longer for the maximum reduction of plasmin in muscle tissue (120 min) in comparison to plasma (30 min) [43].

This could be a first possible explanation for the effectiveness when applied directly into the wound (intramuscular and intraarticular) in comparison to systemic application. Severe trauma often results in pathological hyperfibrinolysis and coagulopathy, i.e. a systemic problem. The majority of hip fractures especially fragility fractures though don`t have a systemic failure in the coagulation cascade. The fracture and surgery lead to a state of local hyperfibrinolysis which needs local treatment and stabilization of existing and forming clots and may be addressed best by topic application, which quickly reaches high activity levels and shows a longer duration. Of the two major incidents leading to total blood loss, fracture itself and surgical procedure, only the latter can really be addressed in hospital. During the combined application where 1 g is applied at the beginning of the surgery plasma levels rise quickly but as seen it takes more time to dissolve and reach relevant levels in the area of interest. By this time the surgical procedures with an average time of 40–120 min for hemi-/total arthroplasty, the second dose has been applied into the wound plasma binding has within 30 min already reached therapeutic levels and by competitive binding blocked some of the activity for the second dose, which may then become less effective and therefore eliminated unchanged.

Our previous findings supported the use of tranexamic acid also for per/subtrochanteric fractures as it has been proven they have a higher overall blood loss [7, 24]. In our current results we see a trend towards reducing blood loss for these fractures, but we were not able to show significant changes. This agrees with Blumenthal et al. [44] who compared geriatric hip fractures treated with arthroplasty to cephalomedullary nailing and concluded that tranexamic acid was only beneficial for arthroplasty procedures and former good results could have been influenced by comparing different fracture morphologies and surgeries altogether. By contrast, Lei [18] and Tengberg [45] both showed a reduced blood loss for extracapsular hip fractures.

Few studies showed a rise in complications focusing on thromboembolic complications. We registered a small increase in complications, which was not significant and no increase of adverse vascular events. Xi et al. [22] recorded comparable general complications rates and Geddes et al. [21] who specifically recorded thromboembolic events could not see an increase after tranexamic acid. Tranexamic acid is not associated with higher infection rates and some studies postulated a protective effect against implant associated infections [46, 47]. In contrast to this we found a higher infection rate after application of tranexamic acid with 4.8% vs. 2.5% which we cannot explain especially as mortality rate was lower after administration of tranexamic acid. The injection into the joint itself should not lead to a higher infection rate as performed during surgery.

The main limitation is the retrospective and unrandomized design of the study. Topic application has been shown to be efficient for hip and knee arthroplasty and we can try to but not fully pharmacologically explain our very good results for single topic application especially when comparing it to combined usage. The strength of the study are the large and equally distributed groups which allow a good comparison of the application methods and the evaluation of blood loss as well as hemoglobin and transfusion rates as multiple outcome parameters. Furthermore, we have to note that whilst all patients with tranexamic acid have no drains there are patients before 2018 in the control group with drains. Analysis comparing drains with no drains for blood loss showed no differences (drain: 1449.3 ml vs. without: 1438.2, p < 0.7) which is consistent with studies showing no difference in blood loss for drains in elective hip surgery [48].

## Conclusion

Tranexamic acid effectively reduces blood loss and transfusion rates and shows higher hemoglobin levels postoperatively, without increasing the risk of thromboembolic events after proximal femoral fractures. We were able to show that single topic application for arthroplasty treatment of femoral neck fractures with arthroplasty seems to have better results for blood loss and number of transfused RBCs than single i.v. or combined application. Generally, the application of tranexamic acid leads to higher postoperative hemoglobin levels. Further investigation into the pharmacokinetic reasons, for example by measuring plasmin levels during surgery are needed.

## Data Availability

The datasets used and/or analysed during the current study available from the corresponding author on reasonable request.
